# *C. elegans*-on-a-chip for *in situ* and *in vivo* Ag nanoparticles’ uptake and toxicity assay

**DOI:** 10.1038/srep40225

**Published:** 2017-01-09

**Authors:** Jin Ho Kim, Seung Hwan Lee, Yun Jeong Cha, Sung Jin Hong, Sang Kug Chung, Tai Hyun Park, Shin Sik Choi

**Affiliations:** 1 Department of Energy Science and Technology, Myongji University, Yongin, Gyeonggi-do 17058, Republic of Korea; 2 School of Chemical and Biological Engineering, Seoul National University, Seoul 08826, Republic of Korea; 3 Department of Mechanical Engineering, Myongji University, Yongin 17058, Republic of Korea; 4 Advanced Institutes of Convergence Technology, Suwon, Gyeonggi-do 16229, Republic of Korea; 5 Department of Food and Nutrition, Myongji University, Yongin, Gyeonggi-do 17058, Republic of Korea

## Abstract

Nanomaterials are extensively used in consumer products and medical applications, but little is known about their environmental and biological toxicities. Moreover, the toxicity analysis requires sophisticated instruments and labor-intensive experiments. Here we report a microfluidic chip incorporated with the nematode *Caenorhabditis elegans* that rapidly displays the changes in body growth and gene expression specifically responsive to the silver nanoparticles (AgNPs). *C. elegans* were cultured in microfluidic chambers in the presence or absence of AgNPs and were consequently transferred to wedge-shaped channels, which immobilized the animals, allowing the evaluation of parameters such as length, moving distance, and fluorescence from the reporter gene. The AgNPs reduced the length of *C. elegans* body, which was easily identified in the channel of chip. In addition, the decrease of body width enabled the worm to advance the longer distance compared to the animal without nanoparticles in a wedge-shaped channel. The transgenic marker DNA, *mtl-2::gfp* was highly expressed upon the uptake of AgNPs, resulting in green fluorescence emission. The comparative investigation using gold nanoparticles and heavy-metal ions indicated that these parameters are specific to AgNPs. These results demonstrate that *C. elegans*-on-a-chip has a great potential as a rapid and specific nanoparticle detection or nanotoxicity assessment system.

Since nanotechnology emerged, it has grown rapidly and expanded into a variety of areas. Nanoparticles have been developed and are manufactured as building blocks or sources of various materials and devices^1^. As nanoparticles have unique physical and chemical properties, they are widely applicable for advanced, electrical, optical, mechanical, and structural purposes, and indeed are now being used in a range of commercialized products^2^,^3^,^4^. Owing to their widespread use, nanoparticles have been released into the environment, and consequently, they are now considered a risk for humans^5^. The properties of nanoparticles differ from their respective bulk components, making it difficult to predict any potential toxicity^6^. While the mechanisms behind the nanoparticles’ toxicity remain unknown^7^,^8^, the development of improved methods for assessing nanoparticle toxicity has become an area of increasing importance^9^,^10^.

In particular, silver nanoparticles (AgNPs), which display beneficial antimicrobial activity, are among the most widely used metal-derived nanomaterials and are found in a wide range of everyday consumer products, including cosmetics, clothing, household items, medical devices, and food packaging^11^,^12^. Due to their prevalence in everyday products, AgNPs have now been released into the environment^4^,^13^ and make a threat to human health. In fact, AgNPs have been shown of late to be toxic in a variety of organisms^1^,^14^,^15^,^16^.

*Caenorhabditis elegans* (*C. elegans*) has the promise of being a useful model organism for assessing nanoparticle toxicity. These animals are free-living, transparent nematodes, ~1 mm in length, that have a life cycle of a few days. They are therefore considerably less complex than mammals and can be easily grown and studied^17^. The *C. elegans* genome has been completely sequenced, and its genetic mechanisms have been studied extensively. Furthermore, there is a high degree of homology between the *C. elegans* genes and the human genes (60–80% homology)^18^,^19^. For these reasons, *C. elegans* has been used as a model system to study the toxic effects of AgNPs. In fact, AgNPs have been shown to be toxic to *C. elegans* in multiple ways, causing protein or DNA damage, oxidative stress, reduced survival, reduced reproductive capacity, and inhibition of growth^20^,^21^,^22^. In many of these studies, immobilization of the worms is required for accurately observing the changes in the organism^23^,^24^. Typically, one of several immobilization methods can be used; worms can be immobilized using glue^25^,^26^, by treatment with sodium azide^27^, or by using anesthetics such as levamisole^28^. Although these methods are well established and broadly accepted, they have limitations, including the potential for the occurrence of changes in the worm’s biochemical state and the potential for toxicity. These methods are also labor-intensive and time-consuming^24^. Therefore, alternative methods are required to overcome the limitations of these immobilization methods.

Microfluidic devices that are capable of handling and immobilizing *C. elegans* have been developed of late, and these devices have been successfully applied in sorting^29^, imaging^30^,^31^ and chemo-sensing^32^,^33^,^34^. In these approaches, the moving worm can be immobilized using one of the available techniques, including the introduction of a gelatinous fluid^35^, compression or restriction by pneumatic valves^36^, or physical restriction using a wedge-shaped clamp channel^24^. Although these microfluidic devices have successfully immobilized moving worms for a variety of purposes, no study to date has used an immobilization strategy in the evaluation of the toxic effects of nanomaterial/nanoparticles^37^,^38^,^39^.

In this report, a novel nanoparticle assessment system based on a microfluidic chip that efficiently and rapidly visualizes the uptake and toxicity of the silver nanoparticles in *C. elegans* has been demonstrated for the purpose of both *in situ* and *in vivo* monitoring of nanomaterials. The system uses AgNPs as representative nanomaterials, and the entry of AgNPs into a chip was detected by measuring the body size, moving distance, and fluorescence intensity of animals. The results were compared with the observation conducted using multi-well plates to emphasize the sensitivity of *C. elegans* chip. Other heavy metal ions and nanoparticles with a similar size were examined using the developed system to determine a selectivity of the animal-on-a-chip.

## Results and Discussion

### Characterization of AgNPs

The size and morphology of the AgNPs dispersed in deionized water were determined by the analysis of the transmission electron microscope (TEM) images. The AgNPs that were observed in the TEM images had a mean diameter of 96.4 ± 35.6 nm and existed as spherical single particles (Fig. S1a,b). The hydrodynamic size distribution (Fig. S1c) and zeta potential (−28.2 ± 2.4 mV) of AgNPs were determined by DLS (dynamic light scattering) measurement. These results indicate that the particles are negatively charged and maintain a distance between themselves in an aqueous solution, suggesting the prevention of self-aggregation of AgNPs. Based on the aforementioned measurements, the AgNPs appeared to be well dispersed with suitable sizes, and they were considered unlikely to cause mechanical damage such as scratches or tears to the worm.

### Detection of AgNPs-induced body growth inhibition

**Figure 1:**
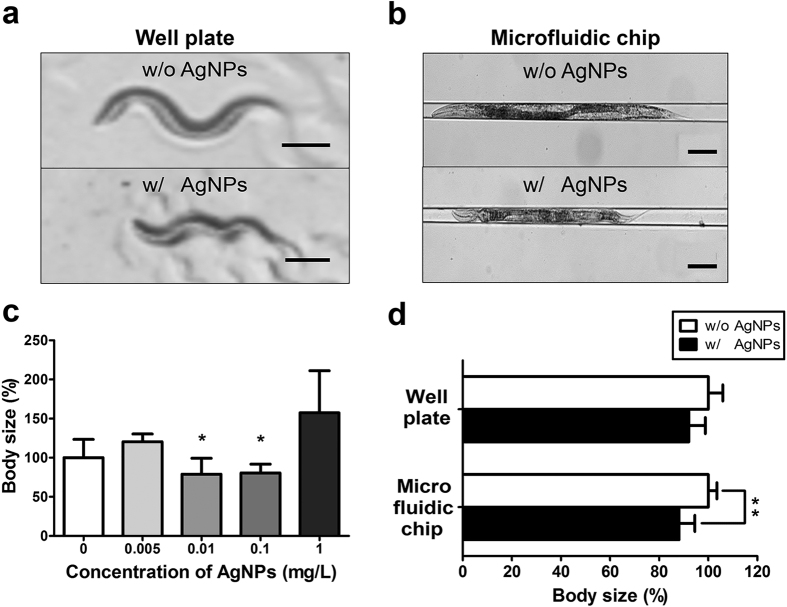
Images of wild-type *C. elegans* (**a**) in a well plate and (**b**) on a microfluidic chip. (**c**) Effect of various concentration of AgNPs on worm body size following 24 h exposure in microfluidic chip. (**d**) Quantitative analysis of worm body size following 24 h exposure of the worm to 0.01 mg/L AgNPs in either a well plate or on a microfluidic chip, respectively. Scale bars are (**a**) 200 μm and (**b**) 100 μm.

The effect of AgNPs on growth inhibition in *C. elegans* has been previously reported^21^. In the study, a measurement of the body size was more difficult due to the continuous movement of the nematode. Therefore, the immobilization of the worm is being employed to obtain an accurate measurement. Many groups have used the anesthetic levamisole to immobilize the worm on an agarose pad, or have used heat-killed worms. Since these methods cause changes in the skin elasticity, however, the accurate determination of the body size change is difficult. Initially, we attempted to obtain a photographic image of the control or AgNPs-exposed worms, without chemical treatment, to determine whether a change in the body size was detectable using image analysis. The body’s wave form, however, caused by the worm’s natural swimming or crawling motion (Supplementary Video V1), has been an obstacle to the accurate assessment of the body size (Fig. 1a).

To precisely measure the body size of *C. elegans*, a microfluidic chip for incubation and immobilization of the individual worm was designed as previously described^23^. Briefly, the chip integrates a single chamber for incubation (Supplementary Video V1) and an adjacent channel for worm immobilization (Fig. S2) (Supplementary Video V2). In this study, the chip was fabricated using the standard soft lithography (Fig. S3)^40^. Using this chip, the body size changes in the control and AgNPs-exposed worms were successfully determined (Fig. 1b). The exposure of the worms to AgNPs resulted in the smaller body size in comparison with the size of worms in the absence of particles. The assessment of the body size in the clamp channel of the microfluidic chip was found to be significantly easier than that in the well plate (Fig. 1a and b).

To investigate the dose-dependent inhibitory effect of AgNPs on growth in *C. elegans*, the wild-type worms in the microfluidic chip were exposed to different concentrations of AgNPs for 24 h (0–1 mg/L), and their body size changes were monitored (Fig. 1c). The 0.01 mg/L AgNPs concentration resulted in the smallest worm size (and hence, the greatest inhibition of worm growth) among all the concentrations of AgNPs. For this reason, this AgNPs concentration was used in the remainder of the studies. In case of the lower (0.005 mg/L) or higher (1 mg/L) concentrations, a statistically significance in the body size inhibition was not found in wild-type *C. elegans* after 24 h of exposure to AgNPs. When the hydrodynamic size of 1 mg/L AgNP suspension was measured using DLS, the z-average diameter of particles was 239.6 ± 33.6 nm at 0 h of incubation. Moreover, the hydrodynamic size of AgNP suspension increased up to 375.6 ± 95.4 nm at 12 h of incubation. The AgNP suspensions are dose- and time-dependently able to form self-aggregates with a large size, which leads to the lower cellular uptake of particles by worms.

In addition to the shorter time and the less labor in the use of *C. elegans*-on-a-chip than multi-well plate, the more precisely measurement of body length led to a statistical significance in the chip system. The relative body lengths of the worms fed with AgNPs were 92.1% of those of control animals without nanoparticles (Fig. 1d). In the microfluidics-based assay, however, the body sizes of the AgNP-exposed worms were found to be 88.1% of those of the control group with a statistical significance (Fig. 1d). These results demonstrate that the body length measurement using a microfluidic chip is more sensitive and accurate than the time- and labor-intensive multi-well plate method.

### Detection of AgNPs-induced longer migration

**Figure 2:**
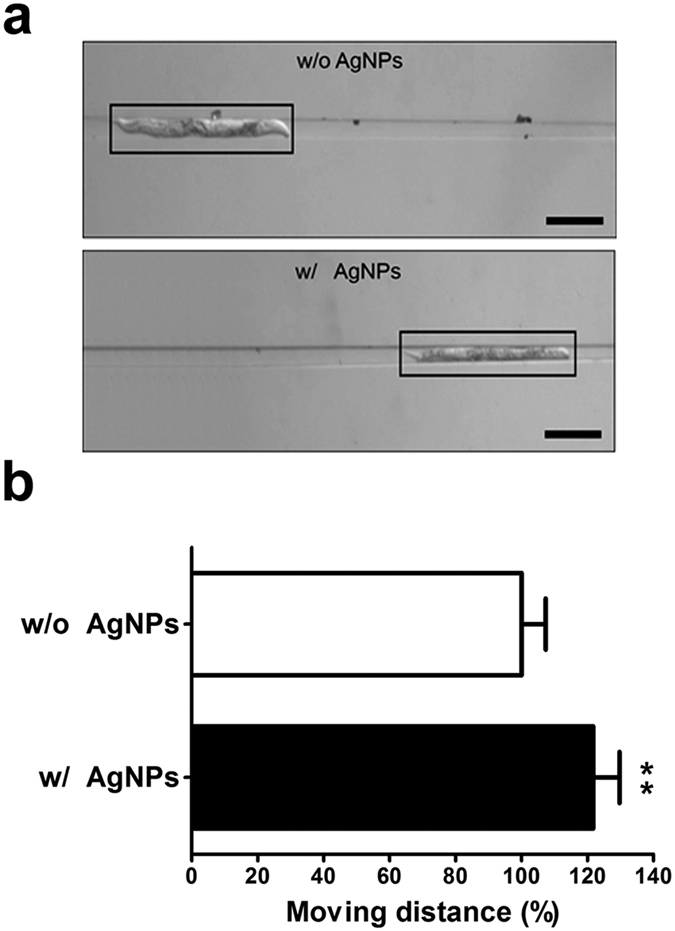
(**a**) Images of wild-type *C. elegans* migration along the wedge-shaped microfluidic chip channel following exposure to 0.01 mg/L AgNPs for 24 h. (**b**) Quantitative analysis of migration distance moved by the worm. Scale bars are 200 μm.

The growth of *C. elegans* involves the occurrence of changes in the body length as well as in the body width. The clamp channel of the microfluidic chip can be used to quantitatively assess the effect of AgNPs on the nematode body width (Supplementary Video V2). The wedge-shaped channel in the clamp chamber gradually decreases in width from 100 to 20 μm (Fig. S2). When a suction force is applied through the outlet reservoir, the distance of migration differs depending on the *C. elegans* body width. The widths of worms that have been exposed to AgNPs are shorter than those of the non-exposed worms. Therefore, after applying a suction force through the outlet reservoir, a worm that has been exposed to AgNPs should migrate further along the wedge-shaped channel compared to a non-AgNPs-exposed worm. Indeed, as shown in Fig. 2a, the images of *C. elegans* migration confirmed that an AgNPs-exposed worm moved farther along the channel than a non-exposed worm.

To quantitate this movement, the distance from the end of the chamber to the head of the worm was measured. The AgNPs exposure allowed the worm to move 20% farther than a non-AgNPs-exposed worm (Fig. 2b). As discussed above, the thickness or width of the worm is one of the key factors indicating the growth inhibition by nanoparticles. Due to the small size and swimming motion of *C. elegans*, however, it is difficult to directly measure the thickness of the worm. The measurement of the distance covered along the wedge-shaped clamp channel of a microfluidic chip under suction force is therefore an alternative method for monitoring the worm thickness, and provides another useful parameter for assessing the AgNPs’ toxicity as well as uptake.

The *C. elegans* develops into its adulthood through embryonic and post-embryonic four larval stages (L1 – L4). Since there is a specific range of body length and width at each developmental stages, both of them are important parameters to evaluate the body growth of *C. elegans*^41^. The adult hermaphrodite *C. elegans* of wild-type N2 have 1250–1400 μm of length and 70–90 μm of width when they grow in the normal culture conditions. However, the L4-stage larvae of *C. elegans* fed with AgNPs shows an abnormal body size in their adulthood with reduction of length or width. Because it is difficult to determine a significant change in body width by measuring the actual size, the length of migration distance has been employed to easily identify the reduction of body width. The wedge-shaped channel enables the difference in the body width of *C. elegans* to be amplified and visualized on the chip.

### Detection of AgNPs-induced specific gene expression

Given that *C. elegans* has a translucent body, the expression of specific genes can be easily visualized fluorescently in transgenic animals containing the DNA construct with a gene of interest fused to GFP (green fluorescence protein)^17^,^42^. Depending on the gene that was selected for analysis, the fluorescence intensity can provide quantitative information about the effect of toxic materials on gene expression^42^,^43^. To assess heavy metal toxicity using *C. elegans*, metallothionein expression has been used as a biomarker^42^. Metallothionein is a small, cysteine-rich protein associated with metal detoxification and sequestration. It has a high affinity for the heavy metals such as Cd, Cu, Zn, and Hg^44^. *C. elegans* has two isoforms of metallothionein, namely *mtl-1* and *mtl-2*^45^. Transgenic strains of *C. elegans* that express GFP under the control of the *mtl-1* and *mtl-2* promoters (*mtl-1::gfp* and *mtl-2::gfp*), respectively, have been developed^42^.

**Figure 3:**
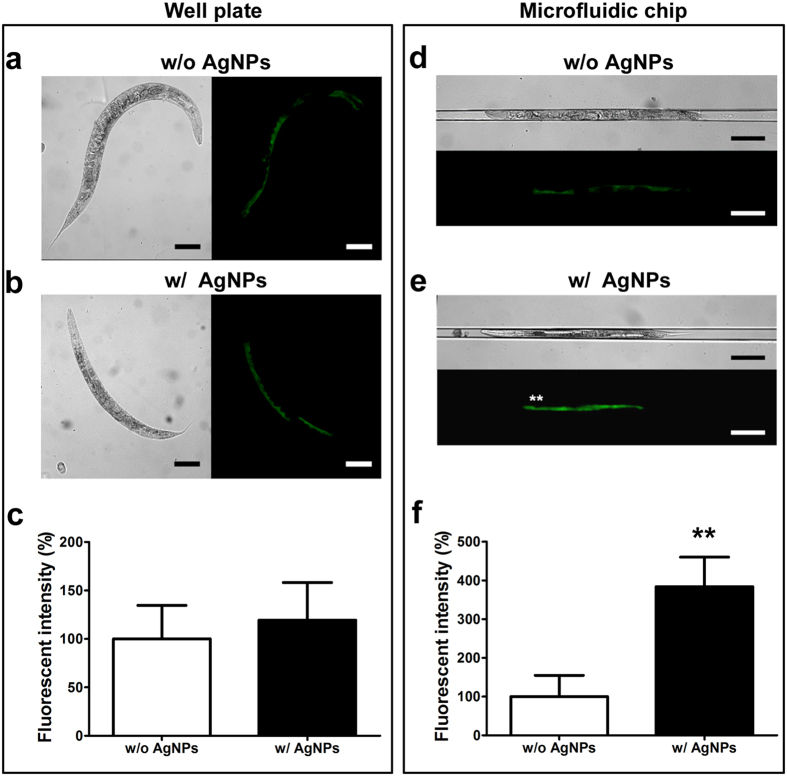
Images of the mutant strain (CL2122 *dvIs15*) after 24 h culture without (w/o) (**a**,**d**) and with (w/) (**b**,**e**) 0.01 mg/L AgNPs. The cultures of *C. elegans* were performed in both well plate (**a**,**b**) and in a microfluidic chip (**d**,**e**). The quantitative measurements of the GFP fluorescence from mutant *mtl-2::gfp* worms cultured on a well plate (**c**) and on a microfluidic chip (**f**). Scale bars are 100 μm.

In this study, the mutant strain CL2122, which contains the *mtl-2::gfp* reporter gene, was used to monitor the uptake of AgNPs and to demonstrate the usability of the microfluidic chip by quantitative measurements of fluorescence intensity. The acute or chronic response of *mtl-2* gene to nanoparticle uptake was not sufficiently reported in *C. elegans* system. However, we found the overexpression of *mtl-2* gene in the wild-type *C. elegans* strain (N2) by real-time PCR analysis (data not shown, unpublished data) after feeding AgNPs. The CL2122 worms were exposed to AgNPs in either multi-well plates or microfluidic chips for 24 h. Based on the real-time PCR analysis, the exposure of *C. elegans* to AgNPs is expected to promote the transcription of the *mtl-2* gene, which can be easily monitored by measuring the GFP fluorescence^46^. The optically transparent nature of PDMS allowed the examination of the fluorescence intensity of GFP in the worm intestine^24^. Figure 3a,b show both the bright field and fluorescence images of the worms in the multi-well plate. When the transgenic worms were exposed to AgNPs in the multi-well plate assay, no significant difference in fluorescence intensity was noted (Fig. 3c). Figure 3d and e show the bright field and fluorescence images of the worms that were exposed to AgNPs in the microfluidic chip assay. The fluorescence signal of the AgNPs-exposed worms increased about four folds compared to the non-AgNPs-exposed worms (Fig. 3f).

These results demonstrate that using this transgenic strain, the proposed microfluidic assay system is more sensitive than the well plate assay in detecting the changes in metallothionein-2 gene expression brought about by AgNPs exposure. Although the precise reason for the increased sensitivity has not been fully understood, it was suggested that the shorter and simple procedure for the sample preparation in the microfluidics system was presumably contributed to the stronger fluorescence signal. On the contrary, the multi-well plate method required the more experimental steps for the sample preparation and mounting worms, which gave an adverse effect on the fluorescence signal from worms fed with AgNPs. As an alternative tool, BioSort (COPAS) has been used to observe the fluorescence signals in worms with an advantage of being able to monitor a large number of worms in a short time^47^. This sophisticated instrument is, however, expensive and known to require a large space and trained operators^47^. However, the microfluidic system proposed herein allows incubation, immobilization, and data collection on a single chip. It is believed that this simplicity allowed the users to observe the effect of AgNPs on metallothionein-2 expression with improved assay sensitivity.

### Rapid monitoring of AgNPs’ toxicity by *C. elegans*-on-a-chip

**Figure 4:**
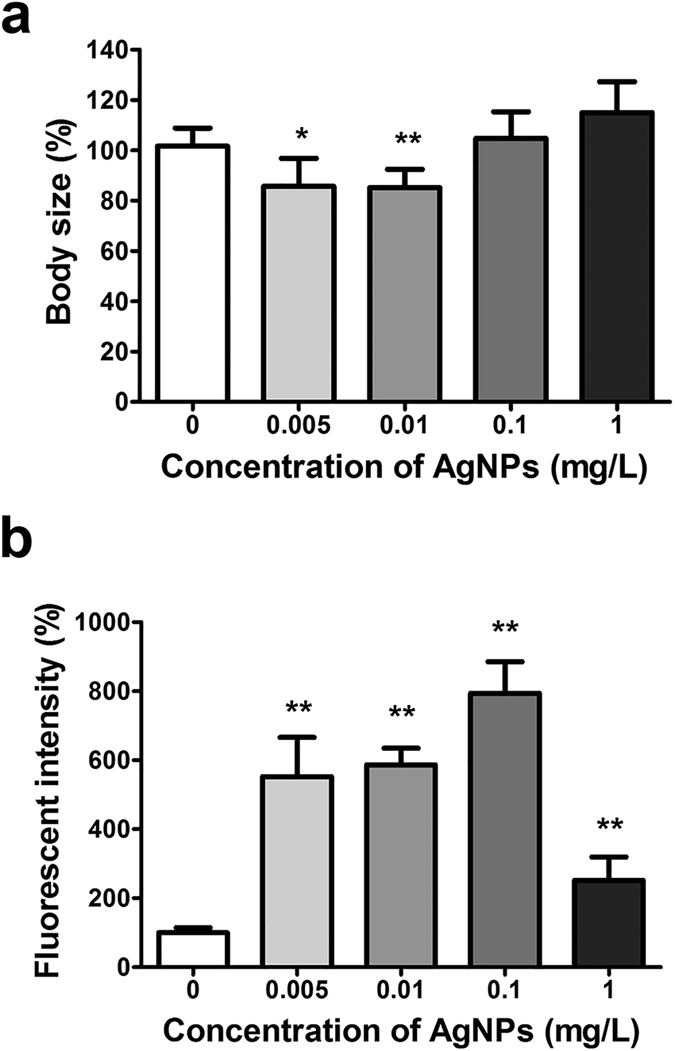
The dose-dependent toxic effect of AgNPs in *C. elegans* after 6 h of exposure to AgNPs. (**a**) Body size in wild-type animals and (**b**) GFP fluorescence signal in the mutant strain (CL2122 *dvIs15*).

In monitoring the toxicity of nanomaterials, an important consideration is that a rapid assessment should be able to be made. Towards this end, the toxic effects of AgNPs on *C. elegans* were investigated in this study at different endpoints. The wild-type (N2) and mutant (CL2122 *dvIs15*) strains were injected into the microfluidic chip. After the exposure of the worms to various concentrations of AgNPs (0, 0.005, 0.01, 0.1, and 1 mg/L) for 6 h, the change in the body size for the wild-type strain and the change in the fluorescence signal for the mutant strain were determined. When the wild-type worms were exposed to AgNPs concentrations ranging from 0.005 to 0.01 mg/L, their body size became smaller than that of the non-AgNPs-exposed worms. It was noteworthy that the growth of the nematode from the L4 stage to the young adult stage was inhibited by such a low concentration (0.005 mg/L) of AgNPs (Fig. 4a). As the AgNPs concentration increased, the effect on growth inhibition actually decreased. This result is potentially explained by the aggregation of the AgNPs at higher concentrations, thus causing a decreased effect of the AgNPs on growth inhibition. In the previous case of 24 h exposure (Fig. 1c), the lower concentration of 0.005 mg/L did not affect the inhibition of body size, whereas only 0.01 mg/L AgNPs shortened the worms’ body length. During the longer period (24 h) of incubation, the inhibitory effect of AgNPs (0.005 mg/L) on body growth was thought to be diminished or overcome because of the smaller AgNPs’ concentration.

The *mtl-2*, a specific gene to AgNPs was significantly overexpressed in the concentration range from 0.005 to 1 mg/L in spite of a short exposure time (6 h) (Fig. 4b). The fluorescence intensity indicating *mtl-2* gene expression increased in the AgNPs’ concentration range from 0 to 0.1 mg/L by dose-dependent manner. However, the highest concentration (1 mg/L) showed less fluorescence intensity than that of the other smaller concentrations. Given that the hydrodynamic size of AgNPs increased over 200 nm at the higher concentration (1 mg/L) by forming self-aggregates, the 1 mg/L AgNPs reduced the onset of *mtl-2* gene expression due to the lower cellular uptake of large particles. However, these results suggest that the proposed system can detect the uptake and toxicity of the very small concentration (5 ppb) of AgNPs at the early time point (6 h) without multiple steps of time- and labor-intensive sample preparation. The overexpression of *mtl-2* gene triggered by the uptake of AgNPs is considered as the earlier process rather than other physiological or phenotypic processes such as the inhibition of body growth, the reproduction of reduction rate and the decrease of survival ratio. Therefore, the fluorescence signal was larger upon incubation for 6 h (Fig. 4b) as compared with 24 h (Fig. 3f). In addition, the L4 larvae fed with AgNPs grew to the young adult and 1-day adult after 6 h (Fig. 4b) and 24 h (Fig. 3f), respectively^48^. The different developmental stage might lead to the difference in fluorescence signal between 6 h and 24 h of incubation time.

### Selectivity of *C. elegans*-on-a-chip

**Figure 5:**
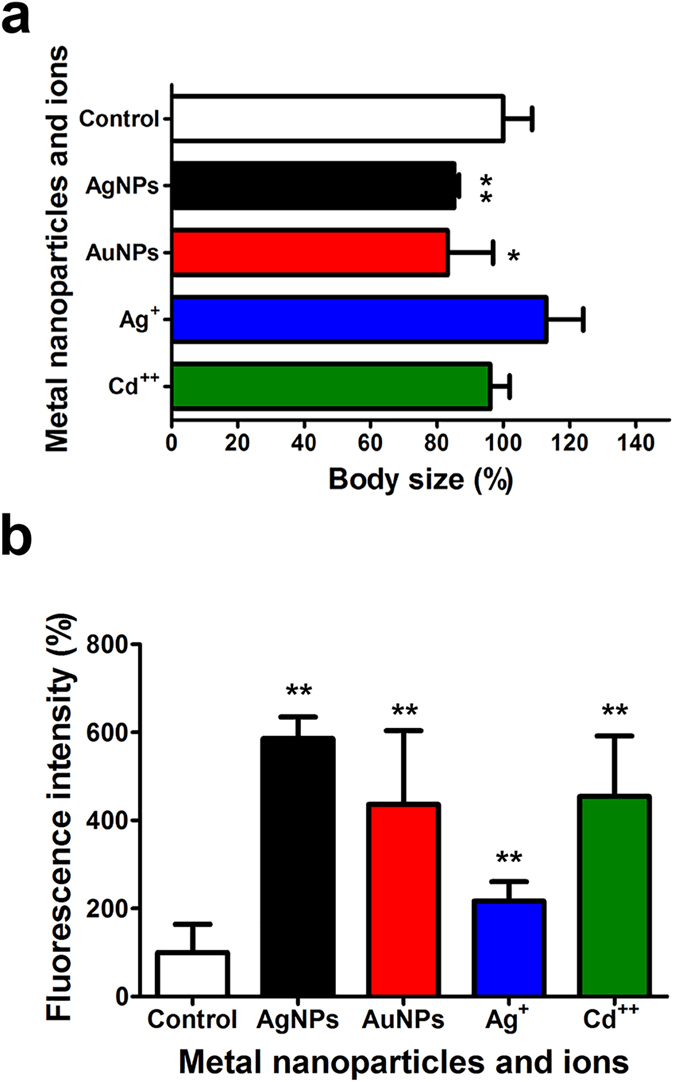
The comparative toxicity assessment on *C. elegans*-based microfluidic chips using metal nanoparticles and ions including AgNPs, AuNPs, Ag^+^ and Cd^2+^. (**a**) Body size in wild-type animals and (**b**) GFP fluorescence signal in the mutant strain (CL2122 *dvIs15*).

To determine whether the microfluidic *C. elegans*-on-a-chip was only specific to AgNPs or responsive to other nanoparticles and ions, AuNPs, Ag^+^, and Cd^2+^ along with AgNPs were prepared and tested using the chip. The changes in the body size and fluorescence signal were monitored using chips containing both the wild-type (N2) and mutant (CL2122 *dvIs15*) strains. Figure 5a,b show the changes in the body size and fluorescence signal that were obtained after the exposure of the worms to the indicated metal ions and nanoparticles. Among the particles and ions, the AgNPs displayed the most significant differences in both body size and fluorescence. Metal ions triggered the overexpression of *mtl-2::gfp* with resulting in the increase of fluorescence, while it did not affect the body growth of *C. elegans*. We have also tested carbon-based nanoparticles including fullerene (nC_60_) and fullerol (nC_60_-OH), however, they did not induce the overexpression of *mtl-2::gfp* on the chips (data not shown, unpublished data).

It was known that AgNPs release Ag^+^ ions in the presence of water, and that the Ag^+^ ions exert a toxic effect on processes such as bacterial cell electron transfer, DNA replication, and oxidative stress^49^,^50^,^51^. In the proposed system, the exposure of worms to Ag^+^ did not affect worm growth, but as expected, it induced an increase in *mtl-2* gene expression. These results indicate that worm growth was inhibited by the AgNPs but not by Ag^+^, whereas the expression of the *mtl-2* gene was affected by both the AgNPs and Ag^+^. The effect of Cd^2+^ on *C. elegans* body growth and *mtl-2* gene expression was also tested using the microfluidic system. The worms that were exposed to Cd^2+^ showed an increase in *mtl-2* expression, as shown by the increase in fluorescence signal; however, the body growth was not inhibited by Cd^2+^.

There are two independent pathways, the body growth inhibition and the metallothionein gene overexpression, in the AgNPs’ toxicology or the regulatory process. Therefore, there is no correlation between the size reduction and the fluorescence signals. The metal ions including Ag^+^ and Cd^2+^ only trigger the overexpression of *mtl-2* gene without a change of body growth, whereas the uptake of AgNP affects both pathways in *C. elegans* due to the co-existence of two faces, the heavy metal property and the nanoparticle characteristic, in the AgNP. The *mtl-2* gene is responsible for the uptake of metals including Ag, and the growth inhibition is a resultant phenotype by the uptake of nanoparticle including AgNP.

The different profile between Fig. 5a,b emphasizes the usability of two parameters, body size and fluorescence intensity, as specific and quantitative indicators for the assessment of AgNPs because the only silver nanoparticle significantly influences both parameters. Given that the concentration (0.01 mg/L) is much lower than that used in the previous studies^52^,^53^, these indicators also possess a sensitivity in addition to the selectivity. These results demonstrate that the *C. elegans*-on-a-chip loaded with N2 and CL2122 strains has a potential applicability for the detection of nanoparticles or assay of their toxicities using multiple parameters including the body size, the migration distance and the fluorescence from a specific gene overexpression.

The proposed *C. elegans*-on-a-chip has a potential as the platform device to determine nanoparticles’ existence and toxicity if the strains with an overexpressed fluorescence protein DNA fused with a gene responsive to the target nanoparticle are adequately selected and loaded on the chip for the target nanoparticle. The overexpressed genes responsive to a specific nanoparticle can be discovered by the help of real-time PCR or DNA chips. Using a chip containing multiple channels entrapped with various types of marker strains, it is also possible to identify or predict what nanoparticles exist in the unknown real sample dropped onto the chip.

## Conclusions

In this study, the microfluidic *C. elegans*-on-a-chip capable of AgNPs detection and its toxicity assay was developed to minimize time, labor and the use of sophisticated instruments. This chip-based assessment allows the incubation, immobilization, and quantitative measurement of *C. elegans* parameters, including growth (worm length and width), behavior (moving distance), and levels of metallothionein (*mtl-2*) gene expression. These parameters indicating the uptake of nanoparticles are more sensitively and accurately obtained on the chip in comparison with the conventional method using multi-well plate or petri dish-based assay. The proposed *C. elegans*-on-a-chip detected the uptake and toxicity of the very small concentration (5 ppb) of AgNPs at the early time point (6 h) without multiple steps of time- and labor-intensive sample preparation. In addition, the only AgNPs led to both the overexpression of *mtl-2* gene and the reduction of body size among the used nanoparticles and metal ions, suggesting a strong selectivity of the chip. These results demonstrated that the animal chip exerts an efficient monitoring performance, with a significant reduction in labor, space, and time. Therefore, the proposed *C. elegans*-on-a-chip has a great potential as a rapid and on-site system for monitoring the uptake and nanotoxicity of AgNPs.

## Materials and Methods

### Chemicals and nanoparticles

AgNPs (SKU: 576832) and AuNPs (SKU: GRF-60-20) were purchased from Sigma-Aldrich (St. Louis, MO, USA) and Cytodiagnostics (Burlington, ON, Canada), respectively. According to the manufacturer’s protocol, the AgNPs (<100 nm) were obtained using PVP as a dispersant with the purity of 99.5% trace metal basis. The reactant free AuNPs (60 nm) were stabilized using citric acid and dispersed in 0.1 mM phosphate buffered saline (PBS) with the purity of 99.0% reactant free. Both AgNPs and AuNPs suspensions were prepared by diluting the particles in deionized water. The hydrodynamic size distribution and zeta potential of the nanoparticles were measured using a dynamic light scattering (DLS) spectrometer (NanoZS, Malvern, UK). The morphology and size of the nanoparticles were analyzed by TEM (transmission electron microscopy, JEM1010-80 kV, JEOL, Japan). The silver nitrate (AgNO_3_, Sigma, St. Louis, MO, USA) and cadmium chloride (CdCl_2_, Sigma, St. Louis, MO, USA) were prepared by dissolving their respective powders in deionized water.

### Fabrication of a microfluidic chip for nanotoxicity assay

The master mold was manufactured by photolithography using photoresist SU-8 2025 (MicroChem Corp., Newton, MA, USA) (Fig. S3). The poly-dimethylsiloxane elastomer (PDMS; Sylgard 184, Dow Corning, Midland, MI, USA) was mixed with its curing agent (10:1). After degassing of the mixture under vacuum, the PDMS/curing agent mixture was poured onto the master mold and baked at 70 °C. The cured PDMS was peeled from the master wafer and punched to form an inlet and an outlet. After cleaning, the PDMS was treated with oxygen plasma and bonded to a glass slide. The diameter of the incubation chamber was 1.5 mm with a depth of 50 μm. The width of the immobilization channel was tapered from 100 μm to 20 μm at a constant depth of 50 μm.

### Maintenance of *C. elegans*

*C. elegans*, including Bristol N2 (Wild-type), and CL2122, dvIs15 (*mtl-2::GFP*) and *Escherichia coli* strain OP50 were obtained from the Caenorhabditis Genetic Center (CGC, at University of Minnesota, Minneapolis, MN, USA). All nematodes were maintained at 20 °C on nematode growth medium (NGM) seeded with OP50 as a food source^7^,^17^.

### Preparation of synchronous *C. elegans*

For the multi-well plate-based assay, the synchronization of worms was achieved using the following process. *C. elegans* eggs were isolated from mature adults using a hypochlorite solution (1.5% NaOCl and 1.5 M NaOH) and allowed to hatch on fresh NGM agar plates with OP50 as a food source^7^,^17^. The synchronized worms were prepared and grown until the middle of the L4 larval stage. For the microfluidic chip-based assay, the 20 mature adults were transferred to a fresh NGM agar plate supplemented with OP50 as a food source, and the plates were incubated at 20 °C to allow egg laying to occur. After 1 h of incubation, all adults were removed from the plate, and the synchronized worms were cultured until the middle of the L4 larval stage. The worms were then loaded into the microfluidic chip by injection through the inlet reservoir.

### *C. elegans* growth assay

**Figure 6:**
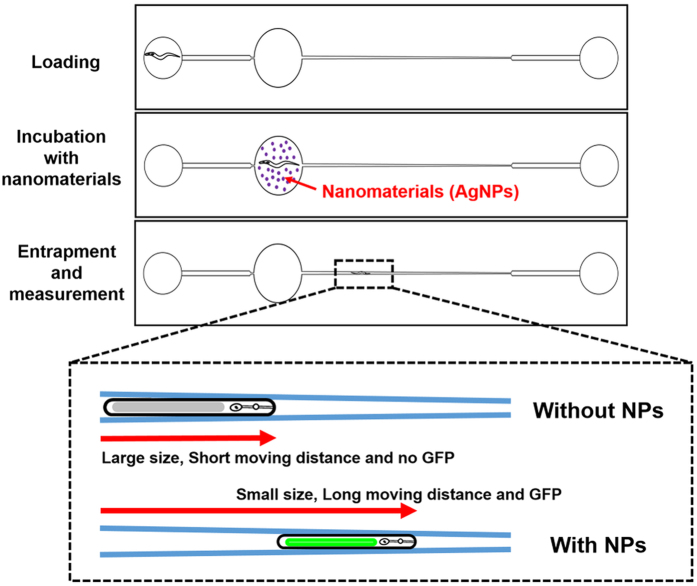
*C. elegans*-on-a-chip to display animals’ body size, moving distance and specific gene expression for Ag nanoparticles’ uptake and their nanotoxicity assay.

For the multi-well plate-based assay, the 10 synchronized L4 stage worms (wild-type, N2) were added to each well. The worms were consequently incubated, in the presence of food, with the 0.01 mg/L nanomaterials for either 6 or 24 h. The nematodes were then rinsed with deionized water and transferred manually to fresh NGM media^17^. For the microfluidic chip-based assay, a single L4 stage worm was injected into the chamber through the inlet reservoir. The worm was incubated with the appropriate nanomaterial for either 6 or 24 h with a food source. The worm was then washed and moved to the clamp channel of the chip by applying a suction force (Fig. 6). The worm’s body size was estimated by microscopy (SZ61, Olympus, Tokyo, Japan) and the resulting microscopic image was analyzed using ImageJ software (http://imagej.nih.gov) 7. Additionally, the distance covered by the worm in the microfluidic chip clamp channel was determined by obtaining microscopic, or photographic images, and analyzing the images. At least 10 replicates were conducted for the precise growth inhibition assay.

### Metallothionein gene expression-related GFP reporter assay

For the multi-well plate-based assay, 10 synchronized (L4 stage) mutant worms (*mtl-2::gfp*) were cultured in the well in the presence of the appropriate nanomaterial in the presence of a food source. After 6 or 24 h exposure, the nematodes were rinsed with deionized water and were treated with 5 mM levamisole solution (as an anesthetic) to immobilize the worm^7^,^17^. For the microfluidic chip-based assay, a single L4 stage worm was injected into the chamber through the inlet reservoir. The mutant worm was incubated with the appropriate nanomaterial for either 6 or 24 h with a food source. The worm was then washed and moved to the clamp chamber of the chip by applying a suction force (Fig. 6). The green fluorescence protein (GFP) fluorescence intensity was monitored using a fluorescent microscope (Axio Imager A2, Carl Zeiss, Jena, Germany) equipped with a Peltier cooled CCD camera. All nematodes were photographed at a fixed fluorescence exposure time. The worms GFP fluorescence intensity was analyzed using ImageJ software^7^.

### Data and statistical analysis

The comparison of experimental data from at least three independent experiments was conducted using a mean value with the error bar (standard deviation, ±S.D.), and the statistical significance was determined by Student *t-test* (Sigma Plot 10.0, SPSS Inc., Chicago, IL, USA). When the *p*-values are less than 0.05 or 0.01, the data are considered statistically significant (*p < 0.05 and **p < 0.01).

## Additional Information

**How to cite this article**: Kim, J. H. *et al*. *C. elegans*-on-a-chip for *in situ* and *in vivo* Ag nanoparticles’ uptake and toxicity assay. *Sci. Rep.* **7**, 40225; doi: 10.1038/srep40225 (2017).

**Publisher's note:** Springer Nature remains neutral with regard to jurisdictional claims in published maps and institutional affiliations.

## Supplementary Material

Supplementary Information

Supplementary Video V1

Supplementary Video V2
