# Supplementary material for: C. elegans-on-a-chip for in situ and in vivo Ag nanoparticles’ uptake and toxicity assay
Source: Sci Rep. 2017 Jan 9;7:40225. doi: 10.1038/srep40225 (PMC5220357; doi:10.1038/srep40225)
Supplement: Supplementary Information [file srep40225-s1.pdf]

Supplementary Information

C. elegans-on-a-chip for *in situ* and *in vivo* Ag nanoparticles' uptake and toxicity assay

Jin Ho Kim[1,†], *Seung Hwan Lee*[2,†], *Yun Jeong Cha*[1], *Sung Jin Hong*[3], *Sang Kug Chung*[3], *Tai Hyun Park*[2,4], *Shin Sik Choi*[1,5,*]

[1]Department of Energy Science and Technology, Myongji University, Yongin, Gyeonggi-do 17058, Republic of Korea, [2]School of Chemical and Biological Engineering, Seoul National University, Seoul 08826, Republic of Korea, [3]Department of Mechanical Engineering, Myongji University, Yongin 17058, Republic of Korea, [4]Advanced Institutes of Convergence Technology, Suwon, Gyeonggi-do 16229, Republic of Korea, [5]Department of Food and Nutrition, Myongji University, Yongin, Gyeonggi-do 17058, Republic of Korea.

[†]These authors contributed equally to this work.

[*]Correspondence and requests for materials should be addressed to S.S.C. (email: sschoi@mju.ac.kr)

1-Sizes and morphologies of silver nanoparticles

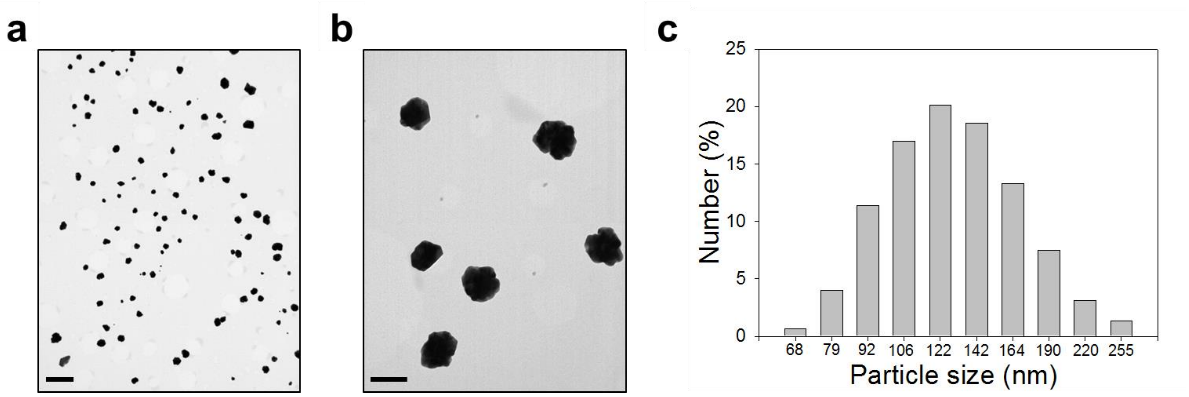

Figure S1. Characterization of AgNPs. The shape and size of AgNPs were analyzed by TEM imaging (a and b). The size distribution of AgNPs were also analyzed by DLS spectrometer (c). Scale bars, 500 nm (a) and 100 nm (b).

2- Incubation chambers and immobilization channels on a microfluidic chip

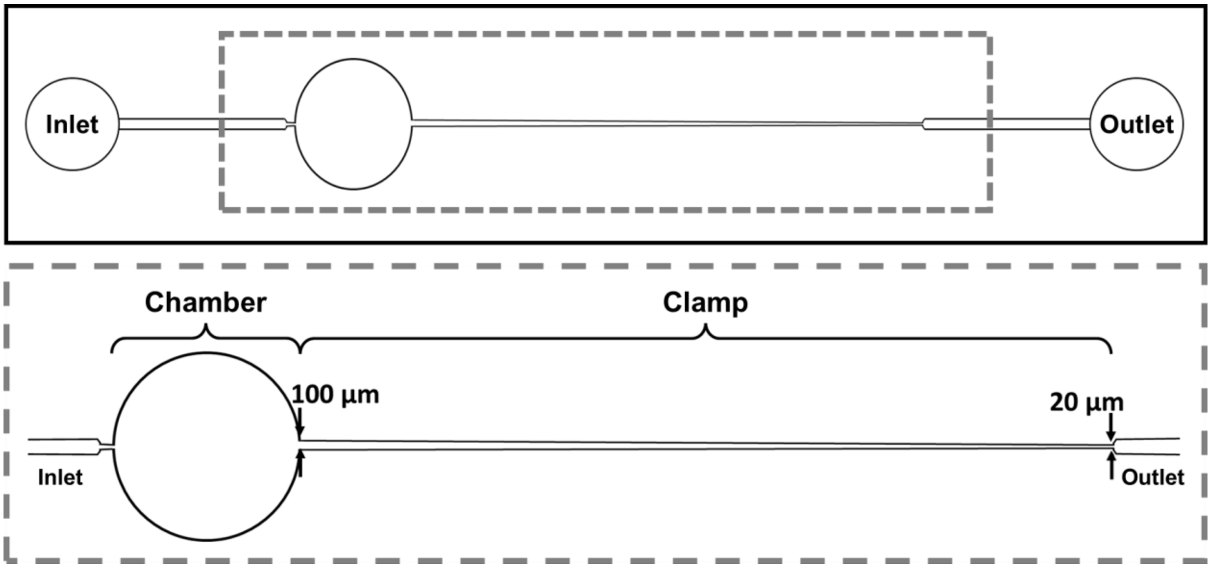

Figure S2. The architecture of microfluidic chip. The microfluidic chip employed a simple design with one inlet/outlet, an incubation chamber and an immobilization clamp. The clamp was designed to contain a tapered shape to aid the immobilization of *C. elegans*.

3- Fabrication process of microfluidic chips

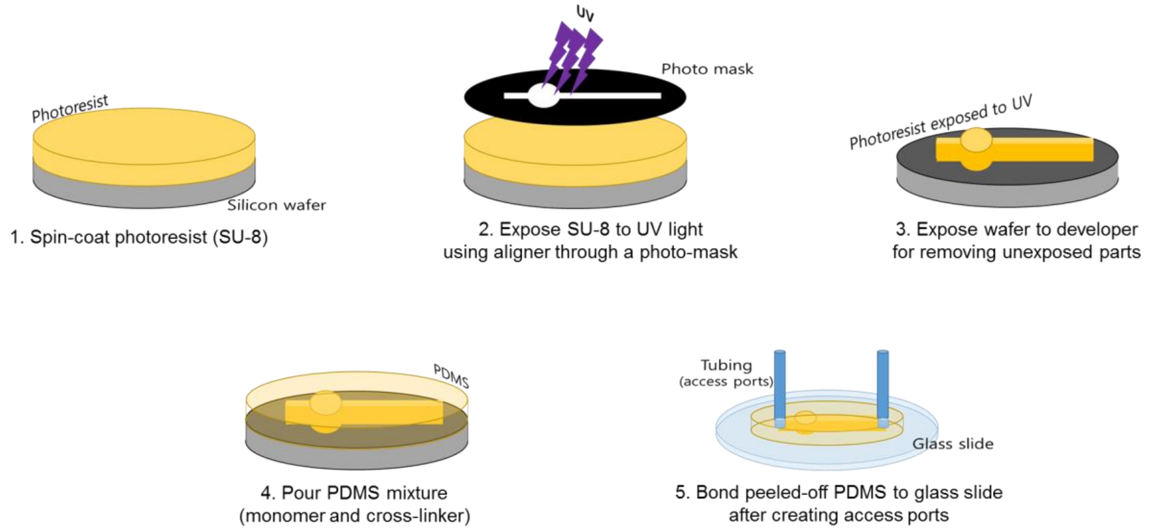

Figure S3. Fabrication of microfluidic chip for the *C. elegans*-based toxicity assay. 1) A negative photoresist (SU-8 2025, MicroChem Corp., USA) was spin-coated onto a 4” silicon wafer (500 rpm 5 sec, 1500 rpm 30 sec) to set the 50 μm height. 2) After pre-baking (65°C 2 min, 95°C 7 min), the photoresist was patterned by using aligner (UV exposure, 15 sec) through a high-resolution sodalime mask. 3) Then, post-baking (65°C 1 min, 95°C 5 min) and development (10 min) were performed to make a master mold for the microfluidic animal chip. 4) The poly-dimethylsiloxane (PDMS; Sylgard 184, Dow Corning, Midland, Michigan, USA) elastomer was mixed with its curing agent (10:1). The PDMS mixture was poured onto the master wafer and baked. 5) The cured PDMS was peeled from the master wafer and punched to form an inlet and an outlet. After cleaning, the PDMS was treated with oxygen plasma and bonded to the glass slide.

Supplementary Video V1. The *C. elegans* swimming in the incubation chamber of the microfluidic chip.

Supplementary Video V2. The *C. elegans* entrapped in the immobilization clamp of the microfluidic chip.
